# Roles of tumor necrosis factor-like ligand 1A in γδT-cell activation and psoriasis pathogenesis

**DOI:** 10.3389/fimmu.2024.1340467

**Published:** 2024-01-29

**Authors:** Shangyi Wang, Mina Kozai, Masaya Hiraishi, Md. Zahir Uddin Rubel, Osamu Ichii, Mutsumi Inaba, Kazuhiro Matsuo, Kensuke Takada

**Affiliations:** ^1^ Laboratory of Molecular Medicine, Faculty of Veterinary Medicine, Hokkaido University, Sapporo, Japan; ^2^ Division of Vaccinology for Clinical Development, Institute for Vaccine Research and Development (IVReD), Hokkaido University, Sapporo, Japan; ^3^ Laboratory of Anatomy, Faculty of Veterinary Medicine, Hokkaido University, Sapporo, Japan; ^4^ Laboratory of Agrobiomedical Science, Faculty of Agriculture, Hokkaido University, Sapporo, Japan

**Keywords:** γδ T cells, psoriasis, cytokine, IL-22, IL-17, TL1A

## Abstract

**Background:**

Interleukin (IL)-17-producing γδT (γδT17) cells mediate inflammatory responses in barrier tissues. Dysregulated γδT17 cell activation can lead to the overproduction of IL-17 and IL-22 and the development of inflammatory diseases, including psoriasis. IL-23 and IL-1β are known to synergistically activate γδT17 cells, but the regulatory mechanisms of γδT17 cells have not been fully elucidated. This study aimed to reveal the contribution of the inflammatory cytokine tumor necrosis factor-like ligand 1A (TL1A) to γδT17 cell activation and psoriasis development.

**Methods:**

Anti-TL1A antibody was injected into an imiquimod (IMQ)-induced murine psoriasis model. TL1A receptor expression was analyzed in splenic and dermal γδT cells. γδT cells were tested for cytokine production *in vitro* and *in vivo* under stimulation with IL-23, IL-1β, and TL1A. TL1A was applied to a psoriasis model induced by intradermal IL-23 injection. Mice deficient in γδT cells were intradermally injected with IL-23 plus TL1A to verify the contribution of TL1A-dependent γδT-cell activation to psoriasis development.

**Results:**

Neutralization of TL1A attenuated γδT17 cell activation in IMQ-treated skin. TL1A induced cytokine production by splenic γδT17 cells in synergy with IL-23. Dermal γδT17 cells constitutively expressed a TL1A receptor at high levels and vigorously produced IL-22 upon intradermal IL-23 and TL1A injection but not IL-23 alone. TL1A exacerbated the dermal symptoms induced by IL-23 injection in wild-type but not in γδT cell–deficient mice.

**Conclusion:**

These findings suggest a novel regulatory mechanism of γδT cells through TL1A and its involvement in psoriasis pathogenesis as a possible therapeutic target.

## 1 Introduction

γδT cells bridge innate and adaptive immune systems through their innate-like potential to rapidly secrete a large amount of cytokines under inflammation (1, 2). γδT cells are particularly abundant in barrier tissues, such as mucosa and skin, and predominate in the early stage of immune responses against microbial infections (2). γδT cells are largely segregated into interferon-γ (IFN-γ) and interleukin (IL)-17 producers functionally committed during intrathymic development. These γδT-cell subsets can be distinguished by the expression or lack of markers, including CD27, CC-chemokine receptor type 6, and IL-1 receptor (IL-1R) (1, 3–6). IL-17-producing γδT (γδT17) cells play crucial roles in the body’s protection against fungi and bacteria, but they are also involved in the pathogenesis of inflammatory and autoimmune diseases as a major source of IL-17 and IL-22 in inflamed tissues (5). It is generally known that inflammatory cytokines IL-23 and IL-1β synergistically induce IL-17 and IL-22 production by γδT17 cells without T-cell receptor (TCR) stimulation (6). In addition to IL-23 and IL-1β, other environmental signals that can activate γδT17 cells have been reported (7, 8). How γδT17 cells sense inflammation to elicit innate function has not been fully elucidated.

Psoriasis is a chronic inflammatory skin disease grossly characterized by inflamed plaques with adherent silvery scales (9). The skin lesions show hyperproliferation and aberrant differentiation of keratinocytes and large neutrophil infiltration. IL-17-producing helper T (Th17) cells in psoriasis pathogenesis have been intensely studied, suggesting a model in which IL-23 derived from dendritic cells and macrophages activates the cytokine secretion of Th17 cells and that Th17-derived IL-17 and IL-22 provoke neutrophil recruitment and epidermal keratinocyte proliferation in the skin (10, 11). In contrast, there is growing evidence that γδT cells contribute to psoriasis development as a primary source of IL-17 and IL-22 in skin lesions (12–15). Deficiency of γδT, but not αβT, cells attenuates IL-17 production and relieves psoriasiform dermatitis in mice intradermally injected with IL-23 (12). Massive infiltration of γδT17 cells has also been found in skin lesions of patients with psoriasis (12).

Tumor necrosis factor (TNF)-like ligand 1A (TL1A) and its receptor, death receptor 3 (DR3), belong to the TNF/TNF receptor superfamily (16). TL1A is initially expressed as a membrane-bound form, and its extracellular domain is released as a soluble protein through cleavage by metalloproteinases (17, 18). TL1A expression is upregulated by stimulation of Fcγ receptors and Toll-like receptors in dendritic cells and macrophages known as major sources of TL1A under inflammatory conditions (19–21). DR3 is expressed in various immune cells, including activated T cells, natural killer cells, and innate lymphoid cells (16), and TL1A-DR3 interaction triggers proinflammatory responses through nuclear factor-κB activation and mitogen-activated protein kinase (17, 21, 22). Genetic studies have revealed the association of TL1A gene (*Tnfsf15*) polymorphisms with psoriasis (23, 24). Elevated TL1A levels in the serum and increased TL1A and DR3 expression in skin lesions have been reported in psoriasis patients (25, 26). Li et al. recently reported that anti-TL1A antibody injection alleviates psoriasis-like symptoms in mice treated with imiquimod (IMQ), indicating the involvement of TL1A in the pathogenesis of this animal model (27). However, the role of the TL1A-DR3 pathway in γδT17 cells and its relevance with psoriasis pathogenesis has not been investigated.

This study reports the crucial role of TL1A in γδT17 cell activation and psoriasis pathogenesis. Anti-TL1A antibody injection inhibited cytokine production by γδT17 cells in IMQ-treated skin. DR3 was constitutively expressed in splenic and dermal γδT cells. TL1A induced cytokine production by γδT17 cells synergistically with IL-23. The effect of TL1A was especially striking in the enhancement of IL-22 production. TL1A exacerbated the symptoms of the murine psoriasis model generated by intradermal IL-23 injection, and TL1A-dependent γδT17 cell activation was crucial in the early pathogenesis of this disease. These findings suggest a novel regulatory mechanism of γδT17 cells through TL1A and provide insights into psoriasis pathogenesis.

## 2 Materials and methods

### 2.1 Mice

C57BL/6 mice were purchased from Japan SLC. TCR γ-chain (Tcrd) knockout (KO) mice (B6.129P2-Tcrdtm1Mom/J) were obtained from The Jackson Laboratory (28). Mice were maintained under specific pathogen-free conditions in our animal facility. All animal experiments were performed under the approval of the Institutional Animal Care and Use Committee of Hokkaido University (Approval no. 20-0172). When data from some independent experiments were accumulated, each experiment was conducted to include all groups consisted of one or more mice per condition. In some experiments, test groups were set to contain more mice than control groups, assuming that the drug treatment produces larger variation among individuals.

### 2.2 Flow cytometry and cell sorting

To prepare single-cell suspensions, spleens were grinded on a metal mesh by a syringe plunger and filtrated through nylon mesh. Skin samples were treated with enzymes as follows prior to the mechanical digestion. Ear pinnae were cut into small pieces and incubated at 37°C for 1 h with 250 μg/mL Liberase TL (Roche Diagnostics) and 1 mg/mL DNase I (Roche Diagnostics) in RPMI 1640 containing 5% fetal bovine serum (Sigma-Aldrich). The single cell suspensions were incubated with antimouse CD16/CD32 (Biolegend) for the blockade of Fc receptors and stained with fluorochrome-labeled monoclonal antibodies (all from Biolegend) against cell surface proteins for 30 min. Intracellular cytokine staining was performed using a fixation and permeabilization buffer set (Thermo Fisher Scientific) according to the manufacturer’s directions. Data were obtained using FACSVerse, LSRFortessa, or FACSAria II flow cytometer (BD Biosciences) and analyzed using FlowJo version 10.3 (TreeStar). To isolate γδT cells, cells were incubated with fluorescein isothiocyanate (FITC)-labeled anti-B220, -TCRβ, -CD11c, -CD11b, and -NK1.1 antibodies (all from Biolegend) and enriched by depleting antibody-bound cells using anti-FITC-conjugated magnetic beads and columns (Miltenyi Biotec). Cells were stained with anti-CD3ε and anti-CD27 antibodies (Biolegend), and CD27^+^ and CD27^−^ fractions in FITC^−^CD3ε^+^ cells were sorted using a FACSAria II (BD Biosciences).

### 2.3 Cell culture

To induce cytokine production, cells were cultured with 20 ng/mL IL-23, IL-1β, and TL1A (all from Biolegend) alone or in different combinations for 24 h. A protein transport inhibitor (Thermo Fisher Scientific) was added during the last 4 h to analyze intracellular cytokines.

### 2.4 Quantitative polymerase chain reaction analysis

Total RNA was extracted from whole ear samples or specific subsets of splenic γδT cells using the RNeasy Plus Micro kit (Qiagen) and reverse transcribed using the PrimeScript RT master mix (Takara Bio). Real-time PCR was performed using TB Green Premix Ex Taq II (Takara Bio) on a LightCycler 96 System (Roche Diagnostics). The primer sequences are listed in **Supplementary Table 1**. Relative mRNA expression was analyzed using the ^ΔΔ^Ct method and normalized to glyceraldehyde 3-phosphate dehydrogenase.

### 2.5 IMQ treatment

Each ear was applied daily with 10 mg 5% IMQ cream (Mochida Pharmaceuticals) or control Vaseline, defining the initial treatment day as day 0. Where indicated, mice were intraperitoneally injected once daily with 20 mg/kg anti-TL1A monoclonal antibodies (clone 5G4.6; Bio X Cell) from day −1 until a day before analysis (29). Single-cell suspensions were prepared from ears on day 3 for intracellular cytokine staining. Ear thickness was measured daily at two specific sites using a micrometer caliper (Mitutoyo) and averaged. Swelling was calculated as changes in the thickness between day 0 and measurement time. Ear pinnae were obtained for histopathological analysis on day 4.

### 2.6 Intradermal cytokine injection

Either side of mouse ears was injected intradermally with 0.5 μg recombinant mouse IL-23 (Biolegend) alone or combined with 0.5 μg recombinant mouse TL1A (Biolegend) in 20 μl volume (30). To analyze IL-17 and IL-22 production, single-cell suspensions were prepared from ears 22 h after cytokine injection. To induce psoriasis-like dermatitis, cytokine injection was repeated daily for 4 days, defining the initial treatment day as day 0. Ear thickness was monitored as described above. Ear pinnae were obtained for histological analysis or qPCR at the indicated time points.

### 2.7 Histological analysis

Ear samples were fixed with 10% neutral buffered formalin. Paraffin-embedded tissues were sectioned and stained with hematoxylin and eosin (H&E). The sections were scanned using a NanoZoomer 2.0-RS virtual slide scanner (Hamamatsu Photonics). Dermis and epidermis thicknesses were measured as described elsewhere with minor modifications (31, 32). The length from the stratum corneum to the basal stratum of the interfollicular epidermis and the length from right under the interfollicular epidermis to the top of the cartilage layer were measured as the epidermis and dermis thicknesses, respectively, using ImageJ software. The dermis and epidermis thicknesses and the number of epidermal layers were measured at eight randomly chosen points in two 600 × 400 μm fields and averaged. For immunohistochemical analysis, deparaffinized sections were treated with 0.3% hydrogen peroxide-methanol. After antigen retrieval, the sections were incubated with blocking 10% goat serum and antimouse cytokeratin-5 (rabbit polyclonal; Biolegend), antimouse proliferating cell nuclear antigen (PCNA)(clone PC10; Calbiochem), or antimouse Gr1 (clone RB6-8C5; R&D Systems) primary antibodies. The sections were incubated with biotinylated secondary antibodies and streptavidin-conjugated horseradish peroxidase (Nichirei). The sections were finally reacted with 0.01% 3,3′-diaminobenzidine. PCNA^+^ and Gr1^+^ cells were counted manually in two randomly chosen 200-μm-long epidermal and dermal areas, respectively, and averaged for statistical evaluation.

### 2.8 Statistical analysis

All data were assessed for normality using Shapiro-Wilk test. Statistical significance was evaluated using two-tailed unpaired or paired Student’s *t*-test, Mann-Whitney U test, or Wilcoxon signed-rank test based on the result of normality test. Analysis was performed with Prism 9.0 (GraphPad Software) and Excel (Microsoft). In all figures, *P*-values < 0.05, 0.01, and 0.001 are shown as *, **, and ***, respectively.

## 3 Results

### 3.1 Involvement of TL1A in γδT-cell activation in IMQ-treated skin

**Figure 1:**
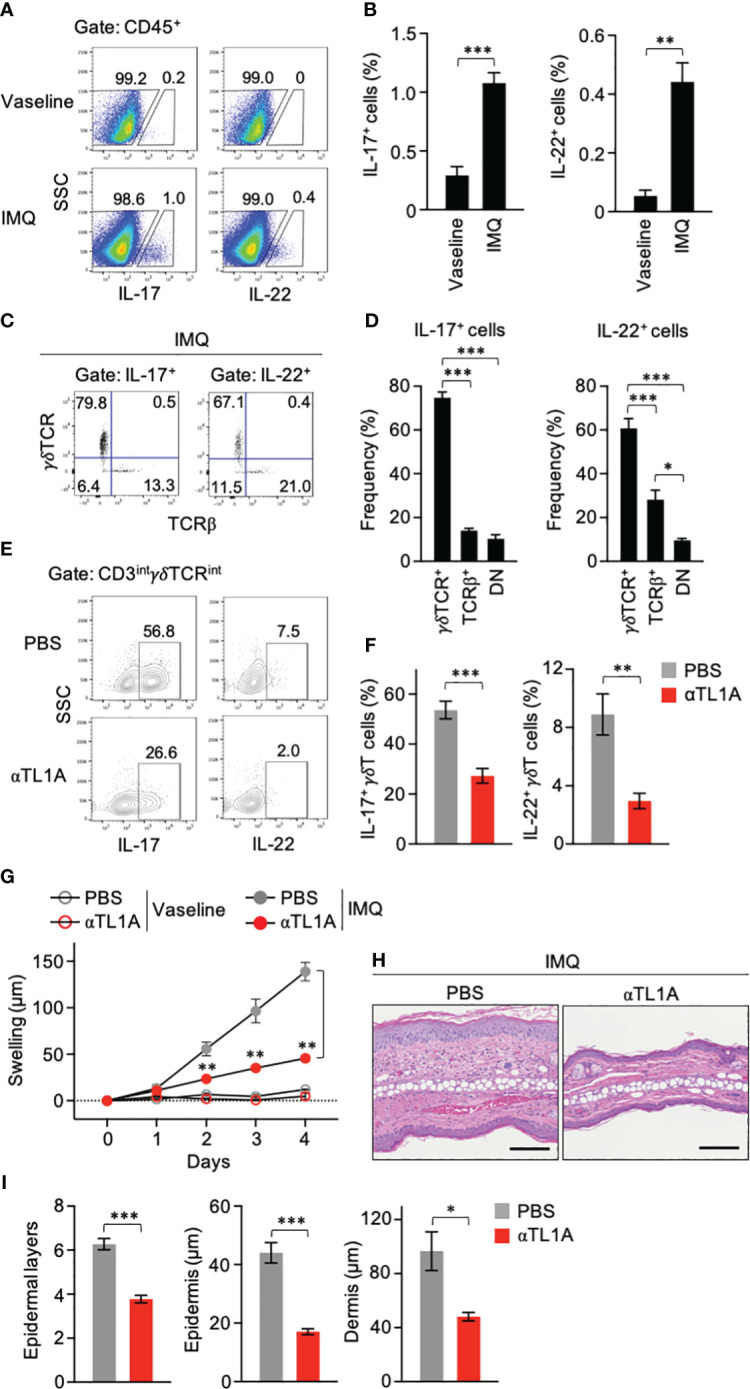
Imiquimod (IMQ) treatment activates dermal γδT cells through TL1A. **(A–D)** Mouse ears were smeared with IMQ cream or control Vaseline for 3 consecutive days. One day after the last treatment, single-cell suspensions from ears were analyzed for cytokine production (n = 5). **(A, B)** Frequencies of cytokine-producing cells in the CD45^+^ gate. **(C, D)** TCR usage of IL-17^+^ and IL-22^+^ lymphocytes in IMQ-treated mice. DN, double negative. **(E–I)** Ears were smeared with IMQ for 3 **(E, F)** or 4 **(G–I)** consecutive days. Anti-TL1A antibodies were injected from day −1 until the day before the analysis. **(E, F)** CD3^int^γδTCR^int^ cells from ears on day 3 were analyzed for cytokine production (n = 5). **(G)** Ear swelling was calculated as changes in thickness from day 0 (IMQ-treated n = 6; Vaseline-treated n = 3). **(H)** H&E staining sections were prepared from ears on day 4. Scale bar, 100 μm. **(I)** Epidermal keratinocyte layers and epidermal and dermal thicknesses were measured in the sections. Representative of three **(A, C, E)** and two **(H)** independent experiments. Accumulated from three **(B, D, F)** and two **(G, I)** independent experiments. Error bars, mean ± standard error (SE). *P < 0.05; **P < 0.01; ***P < 0.001.

Topical application of IMQ, a ligand of Toll-like receptor 7 (TLR7) and TLR8, to the skin induces psoriasis-like manifestations in mice (33), where γδT cells are associated with disease development (12). IL-17- and IL-22-producing lymphocytes were identified in mouse ears after IMQ treatment for 3 consecutive days (**Figures 1A, B**). Remarkably, γδT cells occupied 60%–80% of cytokine-producing lymphocytes in IMQ-treated ears (**Figures 1C, D**). αβT and TCR^−^ cells appeared minor compared to γδT cells (**Figures 1C, D**). These data indicated that γδT cells are the major producers of IL-17 and IL-22 in the skin during the early phase of the IMQ-induced psoriasis model.

Neutralizing anti-TL1A antibodies were injected into IMQ-treated mice. IL-17 and IL-22 production by CD3^int^γδTCR^int^ dermal γδT17 cells (34) was significantly repressed when TL1A availability was limited (**Figures 1E, F**), suggesting the contribution of TL1A to dermal γδT-cell activation in this psoriasis model. Anti-TL1A injection relieved inflammatory symptoms, including skin swelling and cellular infiltrations, after IMQ treatment for 4 consecutive days (**Figures 1G, H**). The number of epidermal cell layers and the epidermis and dermis thicknesses in anti-TL1A-injected mice were also significantly reduced (**Figure 1I**). Notably, the effect of anti-TL1A to ease ear swelling was evident even at the early time point after IMQ treatment for 2 consecutive days when IL-17 and IL-22 are mainly produced by γδT cells (**Figures 1C, D, G**). These data suggested the involvement of TL1A in dermal γδT-cell activation and early disease development in IMQ-induced dermatitis.

### 3.2 TL1A activates γδT17 cells in synergy with IL-23

**Figure 2:**
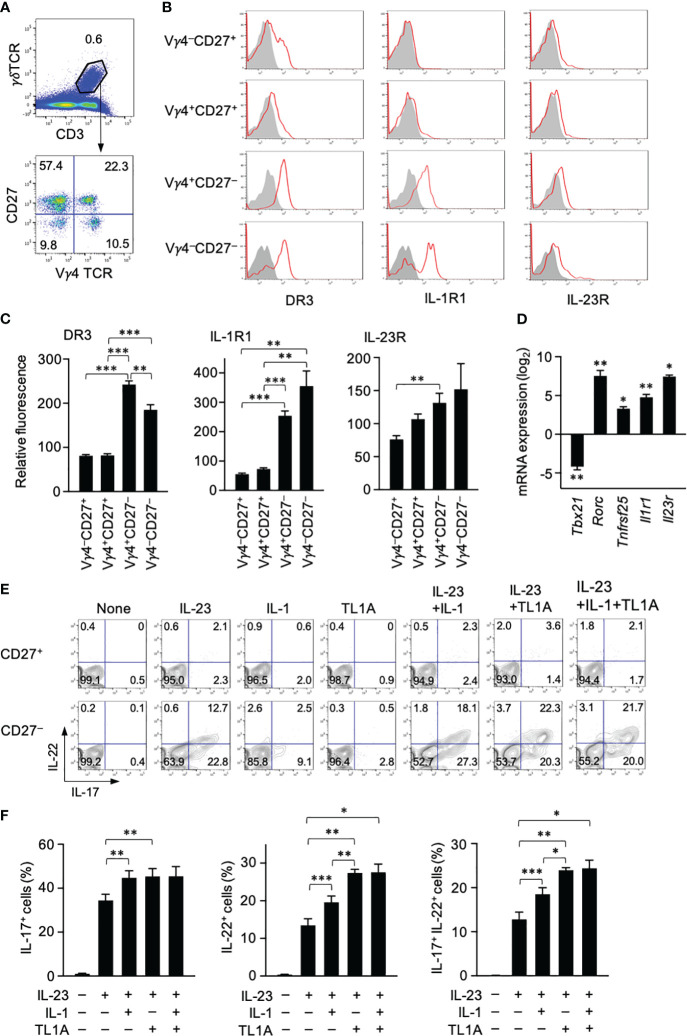
TL1A activates splenic γδT17 cells synergistically with IL-23. **(A)** CD3^+^γδTCR^+^ cells were subdivided into four fractions based on CD27 and Vγ4 expression. **(B)** Expression of DR3, type I IL-1 receptor (IL1-R), and IL-23R in indicated γδT-cell subsets. **(C)** Expression levels of cytokine receptors in each γδT-cell subset are shown as relative fluorescence intensities normalized with the mean fluorescence intensity in total γδT cells defined as 100 (n = 6). **(D)** CD27^+^ and CD27^−^ γδT cells isolated by sorting were analyzed by qPCR for the expression of the indicated genes (n = 3). mRNA expression in CD27^−^ γδT cells is shown as relative levels in CD27^+^ γδT cells defined as 1. The statistical significance of the expression levels between CD27^+^ and CD27^−^ cells is shown by asterisks. **(E, F)** Spleen cells were cultured with the indicated cytokines for 24 (h) A protein transport inhibitor was added for the last 4 (h) IL-17 and IL-22 production was analyzed by intracellular staining. **(E)** Representative flow cytometry profiles of γδT cells at the end of culture. **(F)** Frequencies of IL-17^+^, IL-22^+^, and IL-17^+^IL-22^+^ γδT cells (n = 5). Representative of three **(A, B)** and four **(E)** independent experiments. Cumulative results from three **(C, D)** and four **(F)** independent experiments. Error bars, mean ± SE. *P < 0.05; **P < 0.01; ***P < 0.001.

Expression of DR3, a TL1A receptor, in different γδT-cell subsets was examined using spleen cells from C57BL/6 mice. Splenic γδT cells had four different subsets according to CD27 and Vγ4 expression (**Figure 2A**). CD27^+^ γδT cells are mostly IFN-γ producers, and IL-17 production is limited to CD27^−^ γδT cells (15). γδT17 cells include Vγ6^+^ and Vγ4^+^ cells that can roughly correspond to natural and inducible γδT cells, respectively (6), and Vγ4^+^ γδT17 cells are mainly associated with psoriasis development (35). DR3 expression was apparent in most CD27^−^ γδT cells regardless of Vγ4 expression, whereas only a small fraction of CD27^+^ γδT cells weakly expressed DR3 (**Figures 2B, C**). Type I IL-1R was expressed exclusively in CD27^−^ γδT cells (**Figures 2B, C**). Weak IL-23R expression was detected in all γδT-cell subsets, although CD27^−^ γδT cells tended to express slightly higher levels than CD27^+^ γδT cells (**Figures 2B, C**).

Expression of genes encoding these cytokine receptors was assessed in CD27^−^ and CD27^+^ γδT cells isolated from the spleen. *Tbx21* and *Rorc* encode transcription factors functionally characterizing IFN-γ-producing γδT and γδT17 cells, respectively (6). qPCR analysis verified that CD27^−^ γδT cells express higher levels of *Rorc* and lower levels of *Tbx21* than the CD27^+^ counterpart (**Figure 2D**). As expected, *Tnfrsf25* (encoding DR3), *Il1r1*, and *Il23r* were highly expressed in CD27^−^ γδT cells as *Rorc* whose expression characterizes γδT17 cells (**Figure 2D**). These data suggested that DR3 is preferentially expressed in γδT17 cells in the spleen.

Cytokine production by γδT cells was examined *in vitro* by culturing spleen cells with different combinations of IL-23, IL-1β, and TL1A. Stimulation with IL-23 alone induced substantial IL-17 and IL-22 production by CD27^−^ γδT cells but not CD27^+^ γδT cells (**Figures 2E, F**). Although the response to either IL-1β or TL1A alone was poor, they enhanced cytokine production when mixed with IL-23 (**Figures 2E, F**). Regarding the enhancement of IL-17 production, synergistic effects with IL-23 were similar between IL-1β and TL1A (**Figure 2F**). In contrast, TL1A was significantly stronger than IL-1β in IL-22 induction when used with IL-23 (**Figure 2F**). This striking effect of TL1A enhancing IL-22 production was evident in IL-22^+^ cell frequency (**Figure 2F**) and fluorescence intensity (data not shown).

### 3.3 Dermal γδT cells are ready to respond to TL1A

**Figure 3:**
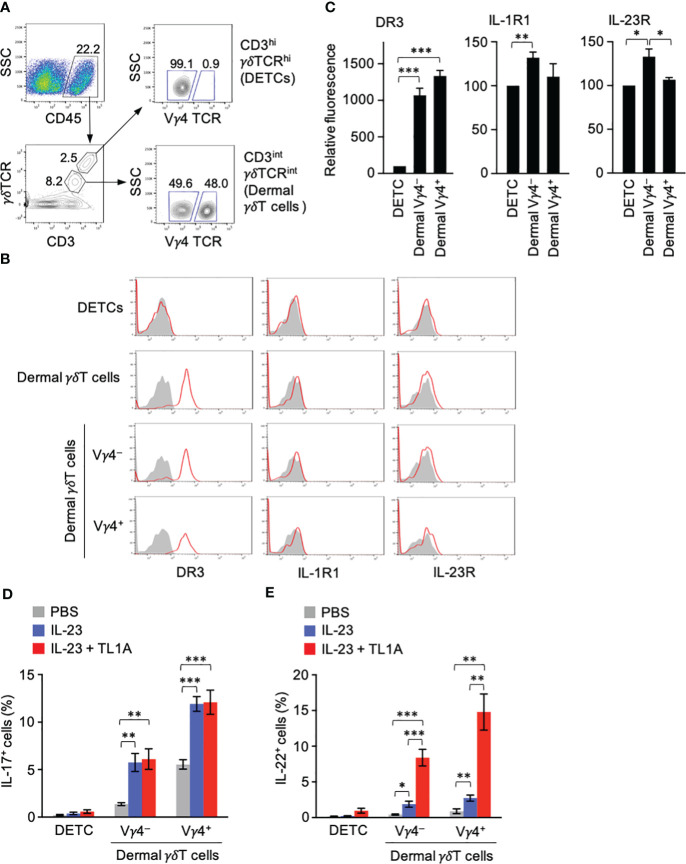
Dermal γδT cells are ready to respond to IL-23 and TL1A. **(A)** CD45^+^ gate in single-cell suspensions from ears contained CD3^hi^γδTCR^hi^ dendritic epidermal T cells (DETCs) and CD3^int^γδTCR^int^ conventional dermal γδT cells. CD3^int^γδTCR^int^ dermal γδT cells were divided into Vγ4^+^ and Vγ4^−^ cells. **(B)** Expression of DR3, type I IL-1R, and IL-23R in DETCs and dermal γδT cells. **(C)** Expression levels of cytokine receptors in Vγ4^+^ and Vγ4^−^ γδT cells are shown as relative fluorescence intensities normalized with the mean fluorescence intensity in DETCs defined as 100 (n = 5). **(D, E)** Mouse ears were intradermally injected with IL-23 alone or combined with TL1A. Twenty-two hours later, DETCs and γδT cells were analyzed for IL-17 **(D)** and IL-22 **(E)** by intracellular staining (n = 7 per group). Representative data of three independent experiments **(A, B)**. Cumulative results from four **(C–E)** independent experiments. Error bars, mean ± SE. *P < 0.05; **P < 0.01; ***P < 0.001.

γδT cells are abundantly present in the skin and provide a barrier against microorganisms. They include γδT TCR^hi^Vγ5^+^ dendritic epidermal T cells (DETCs) and γδTCR^int^Vγ5^−^ γδT cells resident in the dermis (34). The latter, but not the former, γδT cells can produce IL-17 and IL-22 (34). CD3^hi^γδTCR^hi^ DETCs and CD3^int^γδTCR^int^ dermal γδT cells were identified in the single-cell suspension prepared from untreated mouse ear pinnae (**Figure 3A**). DR3 expression was seen in CD3^int^γδTCR^int^ dermal γδT cells but not CD3^hi^γδTCR^hi^ DETCs (**Figure 3B**). Vγ4^+^ and Vγ4^−^ dermal γδT cells expressed DR3 at similar levels (**Figures 3B, C**). Although IL-1R and IL-23R expression in dermal γδT cells was not so clear as DR3, Vγ4^−^ dermal γδT cells expressed slightly higher IL-1R and IL-23R than DETCs (**Figures 3B, C**).

Given the remarkable DR3 expression in dermal γδT cells in a steady state, cytokine production by these γδT cells upon exposure to IL-23 and TL1A *in vivo* was tested. Mouse ears were intradermally injected with IL-23 alone or combined with TL1A, and IL-17 and IL-22 production was examined. DETCs produced little IL-17 and IL-22 in any conditions (**Figures 3D, E**). The overall IL-17 and IL-22 production by dermal γδT cells was predominant in the Vγ4^+^ population and less in the Vγ4^−^ counterpart (**Figures 3D, E**). IL-17 was produced at similar levels upon injection with IL-23 alone or combined with TL1A (**Figure 3D**). IL-22 production was only slightly induced in the group injected with IL-23 alone and remarkably enhanced (more than fourfold) by the combined injection with TL1A (**Figure 3E**). These results suggested an essential role of the TL1A-DR3 pathway in inducing immediate IL-22 production by dermal γδT cells.

### 3.4 TL1A exacerbates the symptoms in the IL-23-induced murine psoriasis model

**Figure 4:**
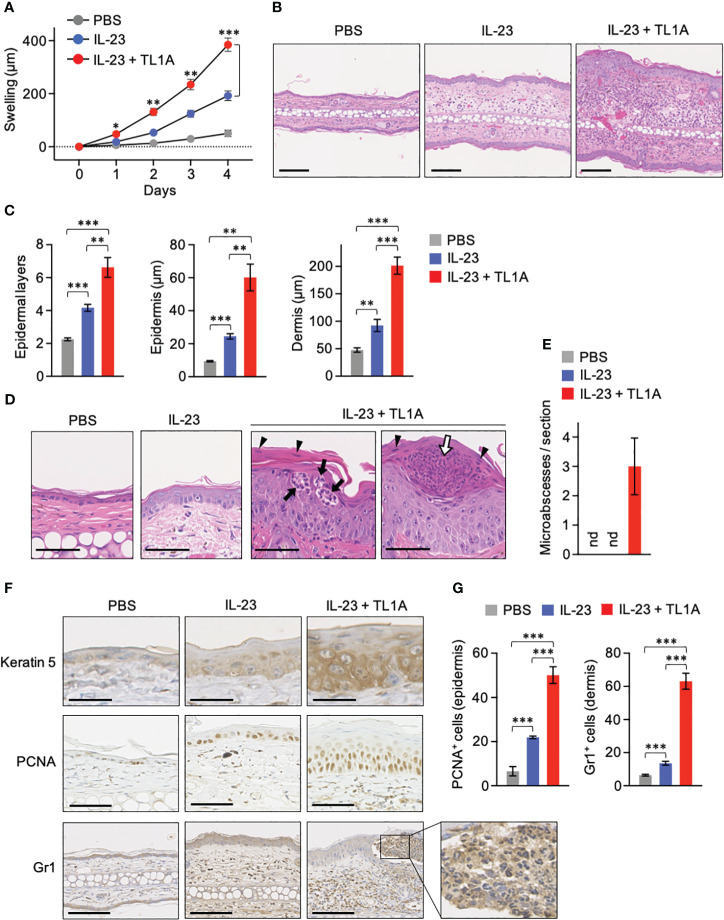
TL1A exacerbates symptoms in the IL-23-induced murine psoriasis model. Mouse ears were intradermally injected with PBS, IL-23 alone, or the combination of IL-23 and TL1A for 4 consecutive days from days 0 to 3. **(A)** Thickness of ears was monitored (n = 4). Swelling was calculated as changes in thickness before the treatments. **(B–E)** H&E staining sections were prepared on day 4. **(B)** Representative view with low magnification. Scale bar, 100 μm. **(C)** Epidermal layers of keratinocytes and epidermal and dermal thicknesses were measured (n = 6). **(D)** Magnified view of the epidermis. Scale bar, 50 μm. **(E)** Number of microabscesses per section (n = 6). nd, not detected. **(F)** Immunohistochemical analysis. Scale bar, 25 μm (top), 50 μm (middle), and 100 μm (bottom). **(G)** PCNA^+^ and Gr1^+^ cells were counted in epidermal and dermal areas, respectively (n = 6). Cumulative results from two **(A)** and three **(C, E, G)** independent experiments. Representative data of three independent experiments **(B, D, F)**. Error bars, mean ± SE. *P < 0.05; **P < 0.01; ***P < 0.001.

IL-23 injection into the mouse skin is a popular psoriasis model that shares various manifestations with human patients, including epidermal hyperplasia (acanthosis), parakeratosis, and cellular infiltration (12, 30, 36, 37). Intradermal IL-23 administration into IL-22-deficient mouse ears has demonstrated the requirement of IL-22 for acanthosis and neutrophil infiltration (36). Given that TL1A was crucial for IL-22 production by dermal γδT cells *in vivo* (**Figure 3E**), how IL-23-induced psoriatic symptoms are affected by the combined administration with TL1A was examined. Mouse ears were injected daily with IL-23 alone or combined with TL1A for 3 consecutive days. As reported, mice injected with IL-23 alone showed overt ear swelling and increased over time (**Figure 4A**). Additional TL1A injection significantly enhanced the response (**Figure 4A**). Notably, this effect of TL1A appeared only a day after the initial cytokine injection (**Figure 4A**), implying the involvement of immune cells, such as dermal γδT cells, ready to respond to these cytokines *in situ*.

Histological analysis revealed that TL1A massively enhances epidermal hyperplasia and cellular infiltration in this psoriasis model (**Figures 4B, C**). In mice injected with IL-23 plus TL1A, epidermal hyperplasia was accompanied by increased epidermal cell layers and hypertrophy of each cell in basal and spinosum layers (**Figures 4C, D**). Enlarged nucleoli indicating robust RNA synthesis was also observed in these epidermal cells (**Figure 4D**). Signs of hyperkeratosis and parakeratosis (remaining nuclei in the thickened stratum corneum as indicated in **Figure 4D**, arrowheads) were obvious after the combined injection with IL-23 and TL1A. These mice also showed neutrophil infiltration into the epidermal layer (**Figure 4D**, closed arrows) and the collection of neutrophils in the stratum corneum (**Figure 4D**, open arrow), similar to Munro microabscesses characteristic of psoriasis patients (38). Microabscesses were absent in all mice injected with phosphate-buffered saline (PBS) or IL-23 alone under the experiment condition that gives the treatment only four times (**Figure 4E**). Keratin-5 expression in suprabasal layers, a hallmark of keratinocyte hyperproliferation in psoriasis (39), was remarkable in mice injected with IL-23 and TL1A (**Figure 4F**). Similarly, PCNA^+^ epidermal cells undergoing proliferation increased in those mice (**Figures 4F, G**). Gr1 signals mainly existed in the reticular layer of the lower dermis in mice injected with IL-23 alone (**Figure 4F**). However, in mice given IL-23 and TL1A, infiltration of Gr1^+^ neutrophils was more intense and extended to the papillary layer of the upper dermis and even into the epidermis, resulting in microabscess formation (**Figures 4F, G**).

### 3.5 TL1A-mediated γδT-cell activation is pivotal for the early development of psoriasis

**Figure 5:**
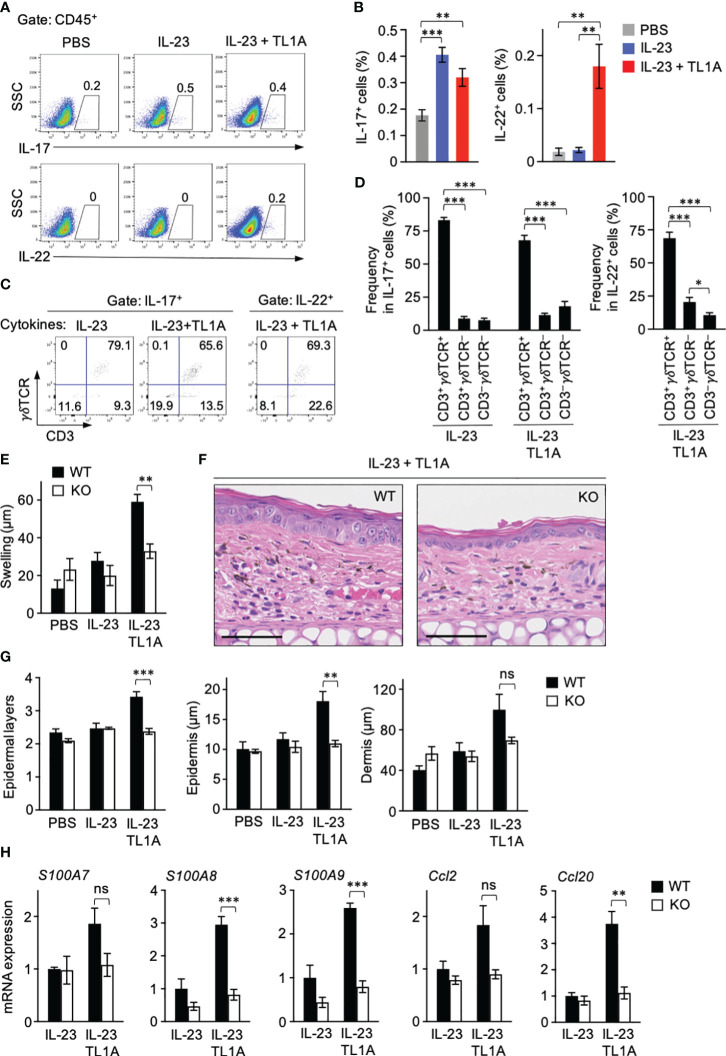
Involvement of TL1A-mediated γδT-cell activation in early psoriasis. Mouse ears were intradermally injected with indicated cytokines. One day later, ears were harvested. **(A–D)** Single-cell suspensions were prepared for flow cytometry analysis (n = 7). **(A, B)** Frequencies of cytokine-producing cells in the CD45^+^ gate. **(C, D)** IL-17^+^ and IL-22^+^ lymphocytes were analyzed for CD3 and γδTCR expression. **(E–G)** Comparison of wild-type and *Tcrd*-knockout mice. **(E)** Ear thickness was measured before and a day after the treatments (n = 4–5). **(F, G)** Histological analysis of H&E sections. **(F)** Representative views from mice injected with IL-23 plus TL1A. Scale bar, 50 μm. **(G)** Epidermal layers and epidermal and dermal thicknesses (n = 4–5). **(H)** Ear samples were subjected to qPCR analysis (n = 4–6). mRNA expression is shown relative to the average values in wild-type mice injected with IL-23 alone defined as 1. Representative of two **(F)** and four **(A, C)** independent experiments. Accumulated from two **(E, G, H)** and four **(B, D)** independent experiments. Error bars, mean ± SE. *P < 0.05; **P < 0.01; ***P < 0.001; ns, not significant.

The disease caused by intradermal IL-23 injection is significantly attenuated in mice deficient in γδT cells compared to wild-type (WT) mice (12). Based on observations that dermal γδT cells were ready to respond to TL1A (**Figure 3**) and TL1A promoted IL-23-induced skin swelling only within a day (**Figure 4A**), the contribution of TL1A-dependent γδT-cell activation to the early inflammation was assessed by analyzing the ear samples one day after single dose cytokine injection. IL-17-producing lymphocytes were detected at comparable levels between the groups injected with IL-23 alone and in combination with TL1A (**Figures 5A, B**). In contrast, IL-22-producing lymphocytes were evident only in the combined injection group at this early time point (**Figures 5A, B**). γδT cells occupied most IL-17- or IL-22-producing lymphocytes (**Figures 5C, D**).

To directly examine the involvement of γδT cells in early disease development, WT and γδT cell–deficient mice were similarly injected with IL-23 alone or combined with TL1A once, and manifestations that appeared within a day were compared. The effect of TL1A enhancing IL-23-induced skin swelling was absent in γδT cell–deficient mice (**Figure 5E**). Histological analysis also showed that γδT-cell deficiency abrogated TL1A-dependent epidermal responses, including the increase of layers and thickness (**Figures 5F, G**). γδT-cell deficiency did not affect dermal thickness at statistically significant levels (**Figure 5G**). Finally, the effect of γδT-cell deficiency on the expression of genes associated with psoriatic skin lesions a day after cytokine injection was examined. Expression of antimicrobial proteins, such as S100A7, S100A8, and S100A9, are induced in psoriatic keratinocytes (40, 41). S100A8 and S100A9 mRNA expression in ear tissues was significantly elevated by the combined cytokine injection compared to IL-23 alone in WT mice, but this TL1A-dependent response was absent in γδT cell–deficient mice (**Figure 5H**). Likewise, the effect of TL1A enhancing the mRNA expression of keratinocyte-derived chemokine CCL20 (42, 43) was γδT cell–dependent (**Figure 5H**). S100A7 and CCL2 mRNA expression showed nonsignificant but similar result (**Figure 5H**). These data indicated that TL1A-dependent γδT-cell activation contributes to the psoriasis development in the very early stage of the disease.

## 4 Discussion

This study demonstrated the involvement of the TL1A-DR3 pathway in γδT17 cell activation. TL1A in cooperation with IL-23, but not by itself, induced IL-17 and IL-22 production. This effect of TL1A was similar to that of IL-1β, a major cytokine regulating γδT17 cell activation (44). However, there was a clear difference between DR3 and IL-1R in their expression pattern. Among γδT-cell subsets in the spleen and skin, IL-1R expression was clearly detected only in splenic CD27^−^ γδT17 cells. This result aligned with the observation in a previous study that IL-1R expression in γδT17 cells varies depending on anatomic sites, including the intestine and the lung (45). In contrast, DR3 was more broadly expressed in various γδT-cell populations distinct in phenotype and anatomic location. Notably, dermal γδT cells in steady state expressed high levels of DR3, suggesting a role of TL1A signaling for the immediate response of dermal γδT cells *in situ*. γδT cell activation through IL-1β has been shown critical for the disease development in IMQ-induced psoriasis model (46, 47). Although IL-1β and TL1A can similarly activate γδT cells, they might act on different γδT cell populations (*e.g.* skin-resident versus migratory) and/or in different stages (*e.g.* inflammation trigger versus progression) during psoriasis development. How these cytokines cooperate for psoriasis development is an intriguing question for future studies.

Intradermal IL-23 and TL1A injection demonstrated that dermal γδT cells strongly depend on TL1A for IL-22 production. IL-22 acts on epidermal keratinocytes and promotes the release of antimicrobial proteins (40, 41). Hence, TL1A-DR3 signaling in skin-resident γδT17 cells may contribute to the host defense by maintaining the barrier against microbial invasion. Additionally, IL-22 also supports the renewal of the skin epidermis by downregulating the molecules associated with the terminal differentiation of keratinocytes and promoting their proliferation (40, 41). IL-22 also has prosurvival effects on intestinal epithelial cells by inducing antiapoptotic genes (48). These regenerative activities of IL-22 suggest that TL1A-dependent dermal γδT-cell activation is important for the homeostatic renewal and wounded tissue repair of the epidermis. The possible role of TL1A and γδT cells in skin homeostasis is an issue that can be addressed in the future.

The function of dermal γδT17 cells immediately producing cytokines in response to inflammation is beneficial for early pathogen clearance, but this characteristic can underlie inflammatory skin diseases. Results showed that γδT cells produce most IL-17 and IL-22 in murine psoriasis models before overt clinical manifestations appear. In the intradermal IL-23-injection model, TL1A significantly accelerated skin swelling only within a day in a γδT cell–dependent manner. A recent study showed that IL-17 derived from γδT cells is primarily required to induce experimental autoimmune encephalomyelitis (49). Therein, the early wave of IL-17 from Vγ4^+^ γδT cells within several hours after autoantigen immunization was critical for recruiting IL-1β-secreting inflammatory monocytes and neutrophils, in turn promoting the priming of pathogenic T cells (49). Likewise, dermal γδT17 cell activation through TL1A during may initially condition the inflammatory environment for subsequent immune responses, including the recruitment of conventional αβT cells. However, whether and how this TL1A-dependent γδT17 cell activation in acute phase of inflammation eventually results in chronic manifestations of psoriasis remains to be clarified. It is possible that TL1A also modulates immune reactions in the later stages of the disease. TL1A exacerbates inflammatory bowel disease by synergistically acting with IL-23 on Th17 cells and enhancing their IL-17 production (50). Similarly, the progression of psoriatic disease with repetitive cytokine injections and IMQ treatments in the experiments may involve the action of TL1A on αβT cells.

IL-17 and IL-22 are cooperatively associated with psoriasis pathogenesis. Deficiency of either of these cytokine signals attenuates but does not fully abolish psoriatic disease development in mice (36, 51). These cytokines are derived from the same sources (γδT17 and Th17 cells) similarly triggered by IL-23. Therefore, upstream factors that differentially activate T cells toward secreting IL-17 or IL-22 have been unclear. Although the involvement of Th22 cells producing IL-22 but not IL-17 has been reported in psoriasis patients (52), the γδT-cell population with such functional characteristics was not observed in this study. In a recent study, anti-TL1A antibody injection was shown to relieve the disease in the IMQ-induced psoriasis model (27). TL1A neutralization represses IL-17 and IL-22 production by γδT cells in IMQ-treated skin. Although anti-IL-17 and anti-IL-17 receptor antibodies are effective for psoriasis (53, 54), the blockade of TL1A-DR3 interaction can also be an alternative therapeutic approach by repressing IL-22 overproduction.

Genome-wide association studies have revealed the link of TL1A gene (*Tnfsf15*) variants with various autoimmune and inflammatory diseases, including psoriasis (23, 24), IBD (55–58), Graves’ disease (59), uveitis (60), Behcet’s disease (61), and systemic lupus erythematosus (62). Increased TL1A levels in the serum or inflamed tissues from patients have also been reported in psoriasis (25, 26), rheumatoid arthritis (63, 64), and IBD (65). The pathogenic roles of TL1A in these diseases have been experimentally demonstrated in animal models or patient lymphocytes (22, 27, 50, 66, 67). Accumulating evidence suggests the role of γδT17 cells in these diseases (12, 44, 68, 69), but the significance of TL1A-mediated γδT17 cell activation in pathogenesis has not been directly shown. Thus, fundamental knowledge from this study can aid in elucidating the pathogenesis of various inflammatory and autoimmune diseases.

In conclusion, this study revealed that TL1A activates γδT17 cells synergistically with IL-23, and this regulatory pathway is associated with the early pathogenesis of psoriatic disease in mice. These findings provide insights into psoriasis pathogenesis and aid in developing therapeutics targeting γδT cells.

## Data availability statement

The original contributions presented in the study are included in the article/**Supplementary Material**. Further inquiries can be directed to the corresponding author.

## Ethics statement

The animal study was approved by Institutional Animal Care and Use Committee of Hokkaido University (Approval no. 20-0172). The study was conducted in accordance with the local legislation and institutional requirements.

## Author contributions

SW: Conceptualization, Data curation, Formal analysis, Investigation, Methodology, Software, Visualization, Writing – original draft, Writing – review & editing. MK: Data curation, Formal analysis, Investigation, Methodology, Software, Validation, Writing – original draft, Writing – review & editing. MH: Investigation, Methodology, Writing – review & editing. MR: Investigation, Methodology, Writing – review & editing. OI: Investigation, Methodology, Writing – review & editing. MI: Resources, Supervision, Writing – review & editing. KM: Funding acquisition, Resources, Supervision, Writing – review & editing. KT: Conceptualization, Data curation, Formal analysis, Funding acquisition, Investigation, Methodology, Project administration, Resources, Supervision, Validation, Visualization, Writing – original draft, Writing – review & editing.
