# Supplementary material for: Roles of tumor necrosis factor-like ligand 1A in γδT-cell activation and psoriasis pathogenesis
Source: Front Immunol. 2024 Jan 29;15:1340467. doi: 10.3389/fimmu.2024.1340467 (PMC10859483; doi:10.3389/fimmu.2024.1340467)
Supplement: Supplementary file 2 [file Table_1.docx]

**Supplemental Table 1. Primers used for qPCR analyses**

| Primer | Sequence |
| --- | --- |
| *Gapdh* F | 5’-TTGTCAGCAATGCATCCTGCAC-3’ |
| *Gapdh* R | 5’-GAAGGCCATGCCAGTGAGCTTC-3’ |
| *Rorc* F | 5’-TGCAAGACTCATCGACAAGG-3’ |
| *Rorc* R | 5’-AGGGGATTCAACATCAGTGC-3’ |
| *Tnfrsf25* F | 5’-CATGTCTGGCAGGTGTGACT-3’ |
| *Tnfrsf25* R | 5’-ACTTTGCCGAGCAGTTCTCA-3’ |
| *Tbx21* F | 5’-GTCCAAGTTCAACCAGCACC-3’ |
| *Tbx21* R | 5’-GTTGGTGAGCTTTAGCTTCC-3’ |
| *Il1r1* F | 5’-TGTGGCTGAAGAGCACAGAG-3’ |
| *Il1r1* R | 5’-CGATCGTCTCATTCCGAGGG-3’ |
| *Il23r* F | 5’-GGCAACATGACATGCACCTG-3’ |
| *Il23r* R | 5’-AGCCCTGGAAATGATGGACG-3’ |
| *S100A7* F | 5’-CCTCGCTTCATGGACACCTT-3’ |
| *S100A7* R | 5’-TTCACCAGCTTGCCCAAGAT-3’ |
| *S100A8* F | 5’-AGGAAATCACCATGCCCTCT-3’ |
| *S100A8* R | 5’-ATCACCATCGCAAGGAACTC-3’ |
| *S100A9* F | 5’-TCATCGACACCTTCCATCAA-3’ |
| *S100A9* R | 5’-TTTGTGTCCAGGTCCTCCAT-3’ |
| *Ccl2* F | 5’-CCCAATGAGTAGGCTGGAGA-3’ |
| *Ccl2* R | 5’-TCTGGACCCATTCCTTCTTG-3’ |
| *Ccl20* F | 5’-CTGCTCTTCCTTGCTTTGGC-3’ |
| *Ccl20* R | 5’-TGGATCAGCGCACACAGATT-3’ |
